# Announcements

**Published:** 2015-05-01

## World Asthma Day — May 5, 2015

May 5, 2015, marks the 17th annual observance of World Asthma Day and the kickoff to Asthma Awareness Month. Asthma is one of the most common chronic diseases in the United States. One in 14 Americans lives with asthma,* experiencing repeated episodes of wheezing, breathlessness, chest tightness, and coughing.

Although asthma cannot be cured, it is possible to manage asthma successfully to reduce and prevent asthma attacks, or episodes. Successful asthma management includes knowing the warning signs of an attack, avoiding things that can trigger an attack, and following the advice of a health care provider.

Members of the public can join experts from CDC and the U.S. Environmental Protection Agency on Tuesday, May 5, at 2:00 p.m. Eastern, for a TwitterChat about asthma, common asthma triggers, and how to create an asthma action plan. To join the moderated conversation, follow @CDCEnvironment on Twitter and use the hashtag *#AsthmaChat2015* in chat messages. No registration is required.

More information about CDC’s National Asthma Control Program and its public and private partners is available at http://www.cdc.gov/asthma.

## Footnotes

* Additional information is available at http://www.cdc.gov/asthma/most_recent_data.htm.

